# Foamy macrophages in atherosclerosis: unraveling the balance between pro- and anti-inflammatory roles in disease progression

**DOI:** 10.3389/fcvm.2025.1589629

**Published:** 2025-05-02

**Authors:** Adil Ijaz, Bhavya Yarlagadda, Marco Orecchioni

**Affiliations:** ^1^ Immunology Center of Georgia, Augusta University, Augusta, GA, United States; ^2^ Department of Pharmacology and Toxicology, Augusta University, Augusta, GA, United States

**Keywords:** atherosclerosis, inflammation, macrophages, foamy, TREM2, Olfr2

## Abstract

Atherosclerosis is a complex immuno-metabolic disease characterized by lipid accumulation and chronic inflammation within arterial walls, leading to cardiovascular events such as stroke and myocardial infarction. Central to the disease are arterial plaques initiated by modified low-density lipoproteins (LDL), particularly oxidized LDL, deposited in the arterial intima. This deposition activates tissue-resident macrophages (TRMs), inducing a lipid-loaded “foamy” phenotype. Additionally, endothelial dysfunction promotes monocyte recruitment, differentiation into macrophages, and further foam cell formation. Foamy macrophages were initially identified as anti-inflammatory but have recently shown dual functionality, possibly depending on the disease stage and phenotype. Recent mouse and human studies also identified subsets of “foamy” macrophages with both pro and anti-inflammatory features. This review examines “foamy” macrophage complex roles and phenotypic diversity in atherosclerosis, emphasizing their potential as therapeutic targets to reduce inflammation and slow disease progression.

## 1 Introduction

Atherosclerosis is a lipid-driven chronic inflammatory condition of the arteries that leads to stenosis of the blood vessels, hence causing cardiovascular complications such as ischemia, stroke, and heart attack, which is a leading cause of death in the western countries (1). Evidence suggests that damage to the integrity of endothelial cells lining the arterial intima due to multiple insults, such as oxidative stress, leads to the infiltration and accumulation of low-density lipoproteins (LDL) in vascular intimal space (2, 3). Excessive reactive oxygen species (ROS) in arterial intima due to high-fat diet feeding and oxidative stress causes oxidation of LDL to transform into oxidized LDL (ox-LDL), a primary driver of atherogenesis (4). The accumulation of ox-LDL and other modified LDLs, such as acetylated LDL, triggers tissue-resident macrophages (TRM), such as aortic intima resident macrophages (Mac^AIR^) to acquire a lipid-loaded phenotype (defined here as “foamy” throughout the manuscript) to clear the lipid-rich microenvironment and restore homeostasis (5). Concurrently, ox-LDL, as well as endothelial dysfunction, drives monocyte chemotaxis into the intimal space, where they further differentiate into macrophages, leading to the formation of additional foam cells in the process of getting rid of excessive cholesterol from the microenvironment (6, 7). Macrophage-derived foam cells play a significant role in shaping subsequent immune responses and atherosclerotic disease progression (8). Thus, it is known that the number of foam cells increases in the lesion as atherosclerosis progresses. Normal blood vessels have indeed few tissue-resident macrophages, most of which reside in the adventitial space (9).

Macrophages are key innate immune cells that respond to pathogens, tissue-derived signals, metabolites, and dying cells using a wide range of sensors (10). They originate early in development (11–15), populating most tissues as tissue-resident macrophages (TRMs), a population conserved from Drosophila to Humans (16–19). TRMs typically persist within tissues, closely interacting with local cells (20). These embryonically derived macrophages differ developmentally and functionally from monocyte-derived macrophages (MDMs), which arise from bone marrow monocytes sharing a common progenitor with dendritic cells, known as the monocyte dendritic cell precursor (MDP) (11, 19, 21–25). Distinguishing TRMs can be challenging due to tissue-specific marker expression. For example, F4/80 labels resident macrophages in the spleen (26) and liver (27), MerTK is found in the liver (28) and lungs (29) macrophages, CD64 along with F4/80 in the aorta (9), whereas TREM2 is specific for macrophages residing in the brain (30) in mice. Similarly, to identify monocyte-derived macrophages, Ccr2, Cd11b^hi^, and Ly6c are commonly used markers (31). Vascular TRM derives from CX3CR1^+^ embryonic precursors with a postnatal contribution from BM-derived monocytes that colonize the arterial adventitia immediately after birth (11, 32). These vascular macrophages persist into adulthood through local proliferation (33). In atherosclerosis, TRM as well as MDMs can acquire a foamy phenotype characterized by the expression of TREM2. These foam cells were initially viewed as anti-inflammatory and pro-resolving (34). However, recent findings show that myeloid-specific deletion of TREM2 cells markedly attenuates plaque progression (35). On the other hand, TREM2 has also been shown to enhance plaque stability and fibrous cap formation in established atherosclerosis (36). These evidences underscore the need to clarify the inflammatory mechanisms driving foamy macrophage behavior.

This review explores the complex and sometimes controversial processes underlying foamy macrophage formation, functions, and phenotypes in atherosclerosis. By dissecting these aspects, we aim to pinpoint possible therapeutic targets in foamy macrophages that can help reduce inflammation and slow atherosclerosis progression.

## 2 Endothelial dysfunction and oxidized LDL

The vascular endothelium plays a crucial role in maintaining blood vessel homeostasis, acting as a gatekeeper that regulates the movement of macromolecules and fluids between the vascular lumen and the surrounding stroma (37). Cholesterol is essential for proper cell function, as it is an integral component of the cell membrane. LDL acts as a chief carrier of cholesterol to cells (38). Under normal physiological conditions, excessive free cholesterol is transported out of the cells to the liver, mediated by high-density lipoprotein (HDL) (39).

The vascular endothelium is critical for maintaining vascular homeostasis by regulating vasodilation, vasoconstriction, and vascular permeability through tightly controlled mechanisms involving endothelin-1, angiotensin II, prostacyclin, and nitric oxide (40, 41). Impaired vasodilation in response to stimuli (e.g., bradykinin) often signals the onset of endothelial dysfunction. Under oxidative stress, reduced nitric oxide production and excessive ROS disrupt normal vasodilation (42). Simultaneously, increased ROS quenches nitric oxide to form peroxynitrite, which further exacerbates endothelial dysfunction (43). This oxidative environment also increases the expression of adhesion molecules such as VCAM-1 and ICAM-1, promoting monocyte adhesion and inflammation (44, 45). Endothelial dysfunction is widely recognized as an early event in atherosclerosis (46, 47).

Following the initial stage, the progression of endothelial dysfunction is characterized by the retention of LDL in endothelial cells, leading to its modification to oxidized LDL (ox-LDL) through the activity of enzymes like NADPH oxidases (NOX), lipoxygenases, xanthine oxidase (XO), myeloperoxidase (MPO), mitochondria reactive oxygen species (ROS) and uncoupled endothelial nitric oxide synthase (eNOS) (48, 49). Elevated levels of ox-LDL further activate the endothelial cells, leading to the expression of surface adhesion molecules that trigger circulating monocyte transmigration into sub-endothelial layers, where they differentiate into macrophages (50, 51). To reduce the elevated levels of ox-LDL at the lesion site, both TRM and MDMs recognize this modified LDL and engulf it via the scavenger receptors such as CD36, lectin-like ox-LDL receptor-1 (LOX-1) and scavenger receptor A1(SR-A1) leading to the formation of foamy macrophages during the initial stage of plaque formation (52, 53). It has been shown that up to 90% of modified LDL uptake by macrophages is mediated by CD36 and SR-A1 (54).

## 3 Macrophage profiling in atherosclerotic plaque

In the atherosclerotic neointima, macrophages play diverse and critical roles, including clearing cholesterol, promoting an anti-inflammatory phenotype that promotes tissue repair and plaque stabilization, as well as driving a proinflammatory microenvironment that favors the progression of atherosclerosis and leads to unstable plaques (8, 55). Because of these divergent roles, macrophages represent an attractive therapeutic target for atherosclerosis, including (but not limited to) inhibiting monocyte recruitment to the lesion site, reducing their proinflammatory phenotype, and increasing their anti-inflammatory responses (8).

### 3.1 Macrophage subsets defined in mouse atherosclerotic plaque

**Figure 1:**
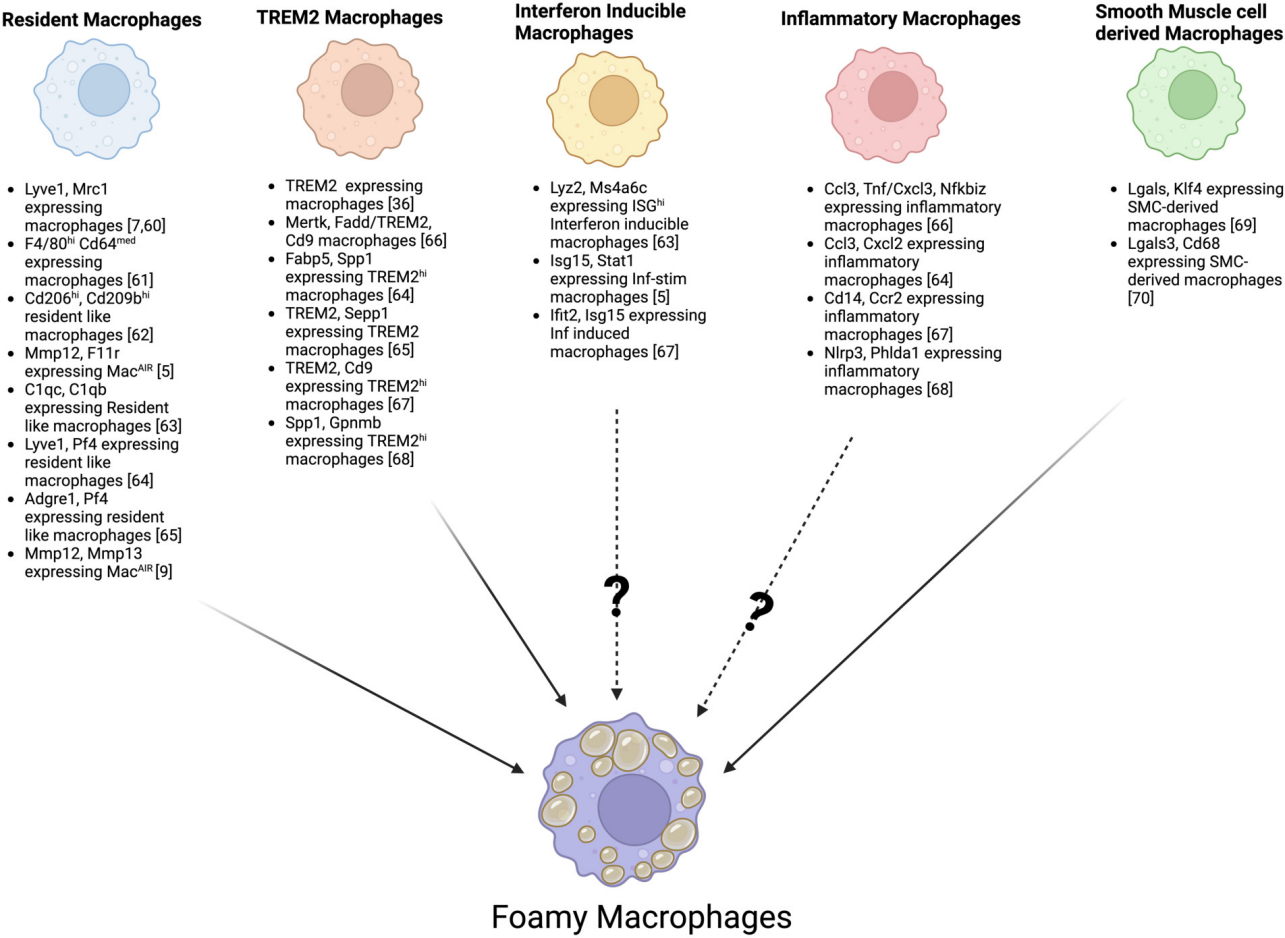
Foamy macrophages possible origin from distinct aortic macrophage subsets based on specific marker genes. Schematic overview illustrating how multiple macrophage subsets, including resident macrophages, TREM2-expressing macrophages, interferon-inducible macrophages, inflammatory macrophages, and smooth muscle cells–derived macrophages, may each give rise to foamy macrophages. Key publications defining various subsets of macrophages based on specific marker genes are shown, highlighting the known and potential transitions (indicated by?) that may lead to lipid accumulation and the “foamy” phenotype.

Due to the initial TRM and the infiltration of MDMs, combined with distinct transcriptional signatures shaped by the microenvironment (including Mac^AIR^), a diverse range of populations of macrophages arises in the plaque. Recent advances in single-cell technologies, such as single-cell RNA sequencing and CyTOF, have greatly improved our ability to identify different macrophage subsets in atherosclerotic plaques. At least 9 phenotypically distinct macrophage subsets with possible distinct functions have been observed in mice (56, 57). These subsets were primarily defined as resident macrophages (Mac^AIR^, and aorta adventitia resident), TREM2 + foamy, interferon-inducible, inflammatory, and smooth muscle-derived macrophages (58). To this extent, multiple independent studies have described a huge heterogeneity in macrophages present in the atherosclerotic plaques and identified various markers to distinguish resident macrophages (5, 7, 9, 59–64), TREM2 (35, 63–67), Interferon inducible macrophages (5, 62, 66), Inflammatory macrophages (63, 65–67), and smooth muscle-derived macrophages (68, 69), all of which may have the ability to transform into foamy macrophages in mouse models of atherosclerosis (Figure 1). Recently, a metanalysis of 12 single-cell RNA sequencing datasets of healthy and atherosclerotic mouse aortas revealed distinct subpopulations with unique transcriptomic signatures within established macrophage groups, such as inflammatory (IL-1β), aortic resident (LYVE1) and foamy (TREM2^hi^) macrophages (56). The authors also observed 2 resident subsets based on CD209low or high expression and 2 foamy (TREM2^hi^) macrophage subsets differentiated based on the expression of *Gpnmb* and *Slamf9*, speculating about possible differences in pro or anti-inflammatory features among them (56). These single-cell transcriptomic analyses of mouse aortas have demonstrated more significant heterogeneity in plaque macrophages than traditional immunophenotyping (6). However, currently, most newly identified macrophage subsets are defined solely by gene expression profiles, lacking comprehensive validation through protein marker characterization and functional analyses.

### 3.2 Macrophage subsets defined in human atherosclerotic plaque

Transcriptional analysis of the macrophage landscape in human atherosclerotic plaques uncovered five distinct macrophage clusters, each exhibiting different functional profiles. Most clusters showed a pro-inflammatory and macrophage activation gene signature, while one cluster demonstrated a foamy transcriptional signature (70). This foamy cluster expressed Apoe, Apoc1, and Plin2—genes involved in lipid uptake, metabolism, and accumulation (70). A recent meta-analysis identified macrophage populations in humans with gene expression patterns similar to those found in mice; notably, key transcripts from the foamy/TREM2^hi^ signature (TREM2, Spp1, Gpnmb, Cd9) defined a distinct macrophage population in human lesions (56).

### 3.3 Foamy macrophages and macrophage-like cells

As discussed, macrophages present in the atherosclerotic plaque may have a foamy or non-foamy phenotype (57). Those that internalize modified LDL or aggregated LDLs via specific receptors become foamy macrophages, whereas those that do not are classified as non-foamy macrophages. Among foamy macrophage phenotypes, Mac^AIR^ are the earliest macrophages to transform into foamy macrophages, even prior to the recruitment of monocytes to the lesion site (5). The origin of foam cells in atherosclerotic plaque has been an interesting topic of discussion. Growing evidence suggests that foam cells are majorly macrophages. However, several studies have shown that also smooth muscle cells and endothelial cells transition into foam cells (71). Thus, multiple studies suggest that almost half of the foam cells may originate from smooth muscle cells (72–74). More importantly, it has been shown that upon taking up modified LDL, smooth muscle cells can even lose their contractile phenotype and express macrophage markers such as CD68, acquiring foamy macrophage-like features (75). Recently, Pan et al. demonstrated by lineage tracing studies that smooth muscle cells can express macrophage-like features and acquire a foamy phenotype in atherosclerotic plaques (76). Li et al. also suggested that smooth muscle cells derived macrophage-like cells may be transient cells as they lose macrophage markers in late atherosclerosis stages (77). This shows that smooth cells-derived macrophages can significantly influence plaque progression and stability. Similarly, macrophages and endothelial cells may also express smooth muscle cell markers (78–80).

The functionality of foamy macrophages may vary according to the macrophage subtype that undergoes this transformation and depending on the disease stage. It is overall accepted that excessive accumulation of foam cells in atherosclerotic lesions leads to the release of matrix-degrading enzymes, tissue factors, and proinflammatory cytokines ultimately creating a necrotic core with a weakened fibrous cap (81). Such unstable plaques are prone to rupture and are a major cause of myocardial infarction and stroke.

## 4 Oxidized LDL uptake and export in foamy macrophages

Macrophages in the arterial intima take up modified LDLs like ox-LDL as part of a lipid homeostasis mechanism, ultimately transforming into foamy macrophages (82). Once internalized, ox-LDL is delivered to lysosomes, where esterified cholesterol is converted into free (unesterified) cholesterol. This free cholesterol is then transported to the endoplasmic reticulum, re-esterified, and stored in lipid droplets (83). The formation of these foam cells is a pivotal event in atherosclerosis, influencing both lesion development and late-stage clinical outcomes such as stroke and myocardial infarction (71, 84). Hence foam cells have emerged as an attractive target to prevent atherosclerosis (85).

Surface receptors primarily involved in the uptake of modified LDL (e.g., ox-LDL) by macrophages include scavenger receptors such as CD36 and LOX1 (86). In addition, foamy macrophages upregulate TREM2, which can govern ox-LDL uptake alongside these scavenger receptors (35). Counterbalancing this internalization process, macrophages can export cholesterol via specialized efflux pathways. ATP-binding cassette (ABC) transporters, particularly ABCA1 and ABCG1, mediate the transfer of cholesterol and phospholipids onto lipid acceptors like apolipoprotein A-I (ApoA-I) and high-density lipoprotein (HDL) (87). The expression of these transporters is tightly regulated by nuclear receptors such as the liver X receptor (LXR), which responds to elevated intracellular oxysterol levels (88) and peroxisome proliferator-activated receptors (PPAR)-gamma (89). Conversely, aberrant downregulation or functional impairment of LXR and ABC transporters can tilt the balance toward pathological lipid accumulation and inflammation (90, 91). Several studies have demonstrated that cholesterol transporters like ABCA1 are crucial for preventing foam cell formation (92). However, findings in this area are not entirely consistent. For instance, mice lacking ABCA1 and SR-B1 exhibit hypocholesterolemia and foam cell accumulation but do not develop atherosclerosis (93). Meanwhile, upregulation of ABCA1 in LDLR knockout mice has been shown to exacerbate atherosclerosis (94). Thus, the precise role of these transporters in atherosclerosis remains somewhat controversial. Overall, it's clear that when cholesterol transporters are downregulated, intracellular ox-LDL accumulates, sustaining the foamy phenotype of macrophages.

## 5 Role of foamy macrophages in atherosclerosis

**Figure 2:**
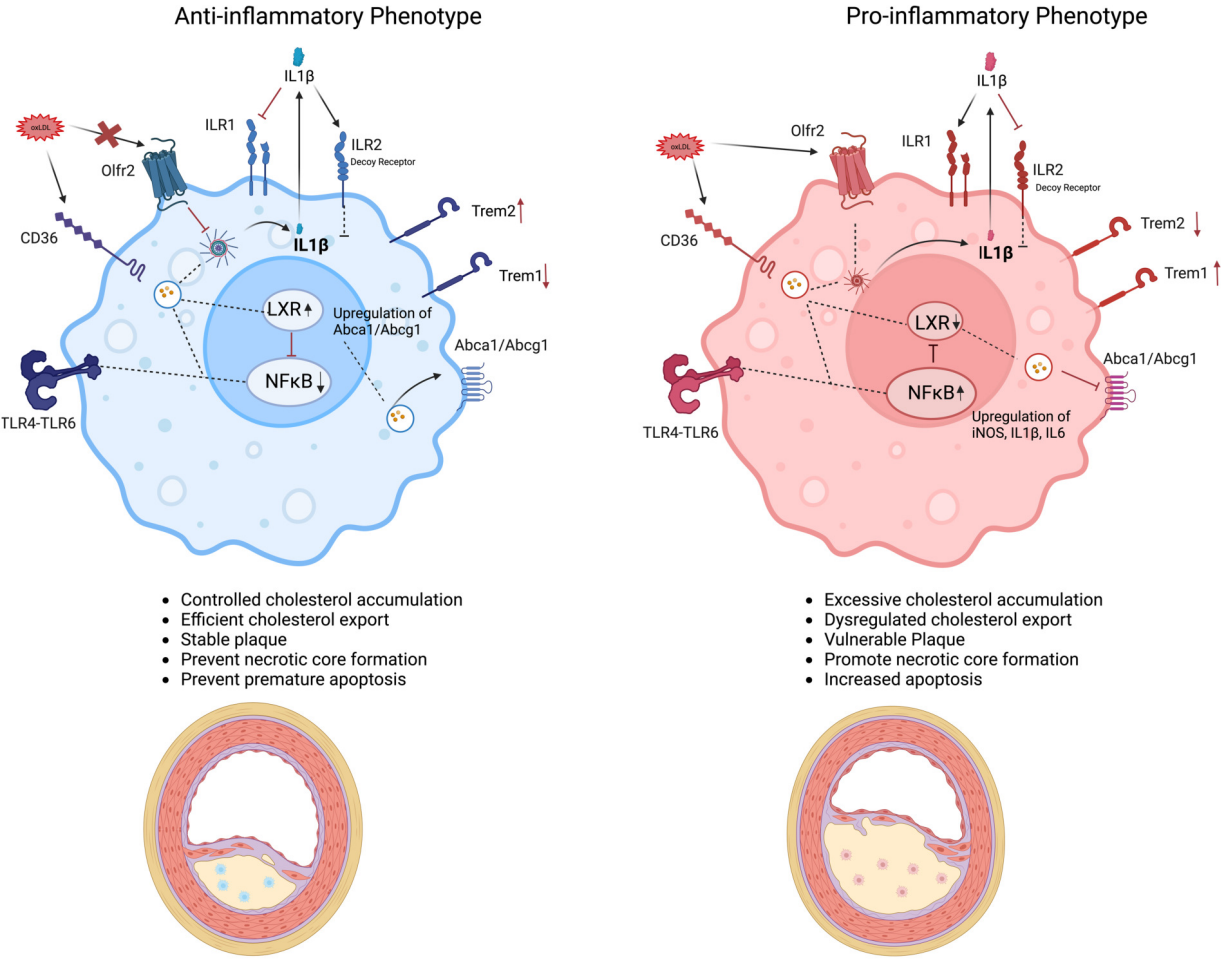
Drivers of foamy macrophage function and phenotype in atherosclerosis. Schematic representation of distinct macrophage foamy phenotypes within the atherosclerotic plaque environment. The anti-inflammatory foamy phenotype (left, blue) maintains controlled cholesterol accumulation, efficient cholesterol export, and reduced inflammatory signaling particularly IL-1β through increasing the expression of IL-1R2 (a decoy receptor) as well as having a dampening effect on NF-κB pathway, and maintaining higher anti-inflammatory gene signature especially IL10 contributing to plaque stability. The anti-inflammatory phenotype is also proposed to have a higher TREM2 expression and lower TREM1 expression contributing to the anti-inflammatory gene signature. In contrast, the pro-inflammatory foamy phenotype (right, pink) is marked by excessive cholesterol accumulation, dysregulated cholesterol export, and heightened production of inflammatory mediators like IL-1β and other inflammatory mediators like iNOS due to activation of NF-κB pathway and reduced expression of TREM2, promoting plaque progression and necrotic core formation. Key molecular pathways (e.g., NF-κB, LXR, TREM2, Olfr2) are highlighted, illustrating how foamy macrophage polarization and the interplay between these pathways can influence atherosclerotic lesion outcomes.

The foamy transformation not only alters the macrophage phenotype but also reprograms their functionality. Internalization of ox-LDL via the scavenger receptors CD36, SR-A1, and LOX1 (95) and possibly its signaling through other receptors like TREM2 and Olfr2 (96), ultimately activates downstream pathways that can either promote an anti-inflammatory, pro-resolving response by enhancing cholesterol clearance and efflux or trigger a pro-inflammatory response through NF-κB and inflammasome activation, thereby inducing IL-6 and IL-1β production and exacerbating inflammation and disease progression (97). IL-1β is a crucial cytokine involved in the progression of atherosclerosis, as highlighted by findings from the CANTOS trial (98). Produced through inflammasome activation, IL-1β is released during pyroptosis and primarily signals through the receptor IL-1R1, activating downstream NF-κB pathways that amplify inflammation (99). Alternatively, IL-1β can bind IL-1R2, a structurally similar decoy receptor expressed by anti-inflammatory foamy macrophages, thereby limiting inflammatory responses (100). Impaired efferocytosis and the activation of multiple cell death pathways, including apoptosis (mediated by activation of caspases 3, 7, and 9, resulting in DNA fragmentation) (101), dysfunctional autophagy (which disrupts lipid homeostasis and promotes cell death) (100), and pyroptosis (mediated by caspases 1 and 11) (102), can further contribute to the proinflammatory foamy phenotype ultimately increasing necrotic core size and plaque instability (103). Figure 2 illustrates how ox-LDL interaction with some macrophage surface receptors may lead to its export or intracellular storage, ultimately influencing the inflammatory phenotype and disease progression.

### 5.1 TREM2 expressing foamy macrophages

Foam cells, especially foamy macrophages, express several surface receptors that can modulate ox-LDL processing. One such receptor is TREM2, a transmembrane receptor belonging to the immunoglobulin family that is expressed on myeloid cells (104, 105). TREM2 is associated with multiple disease conditions and functions, including diabetes, apoptosis, central nervous system dysfunction, and inflammation (106–109). Previous studies using scRNA-seq analysis have demonstrated the expression of TREM2 in CD45 + cells isolated from aortic plaques of atherosclerotic-prone mice (both *Ldlr^−/−^* and *Apoe^−/−^*) (7, 59). TREM2 is particularly expressed in macrophages in the plaques and it is associated with foamy macrophage formation and differentiation (35). It has been shown that the deficiency of TREM2 in macrophages increases the phosphorylation of PPARγ, leading to its decreased transcriptional activity, which subsequently lowers transcription and surface expression of CD36, ultimately decreasing ox-LDL uptake and foam cell formation (110). Similarly, conditional deletion of TREM2 in macrophages reduces atherosclerotic plaque size and en face lesions in mice, negatively impacting foamy macrophage survival and proliferation through increased ER stress and impaired LXR signaling showing the pathogenic role of TREM2 in promoting atherosclerosis (35). However, another independent manuscript described that TREM2 function can stabilize the plaque in late stage of the disease by limiting necrotic core formation, promoting ox-LDL uptake, and enhancing foam cell survival through increased efferocytosis, suggesting the protective role of TREM2 in plaque progression (36). This dual function of TREM2 + foam macrophages could be explained by the recently described different subsets of TREM2 + macrophages that may play diverse functions during atherogenesis (56, 57). These TREM2-expressing aortic foamy macrophages were defined as TREM2^hi^Slamf9 and TREM2^hi^ Gpnmb in atherosclerotic *Ldlr^−/−^* mice (36, 56). By discussing differential gene expression profiles as well as proportions, the authors proposed that TREM2^hi^Slamf9 macrophages show a pro-inflammatory profile characterized by elevated expression of *CD72*, *Ch25h*, *Tnf*, and *Il1b*. On the other hand, TREM2^hi^ Gpnmb macrophages possess a specialized gene signature, including *Gpnmb* and *Fabp5*, suggesting an osteoclast-like differentiation and possible macrophage fusion (56). Interestingly, TREM2 may control these phenotypes as TREM2 deficient foamy macrophages have a reduced expression of scavenger receptors (*CD36*, *Msr1*), foam cell markers (*Gpnmb*, *Spp1*, *Cd5L*), and antioxidant heme oxygenase (*Hmox1*), and efferocytosis compared to TREM2^+^ foamy macrophages suggesting a role of TREM2 in lipid uptake, and foaminess in foamy macrophages (36). Overall, these data show that foamy macrophage populations in atherosclerosis have conserved yet functionally specialized transcriptional states. However, their distinct roles in disease progression still need to be established.

### 5.2 Strategies to lower lipid uptake and foam cell formation

Several surface or intracellular receptors or proteins have been described as either reducing or increasing foam cell formation, thereby mitigating or exacerbating the impact on atherosclerosis. Deleting these receptors or intracellular proteins primarily affects scavenger receptor expression or function, ultimately influencing lipid uptake. Li et al. have shown that ablation of the pyrimidinergic receptor P2Y6 in macrophages limits foamy macrophage formation by lowering SR-A expression and ox-LDL uptake in a mouse model (111). In another study, the macrophage-specific deletion of NFATc3 (nuclear factor of activated T cells cytoplasmic 3) promoted foam cell formation by enhancing ox-LDL uptake via CD36 and SR-A1 in mice (112). Moreover, genetic or pharmacological inhibition of Gsα, G protein stimulatory subunit α, has been shown to decrease atherosclerosis progression in mice by downregulating CD36 and SR-A1 expression (113). A recent study has described the role of a ubiquitin enzyme, USP9X, in suppressing lipid intake in macrophages both in rodents as well as humans (114). USP9X deficient macrophages exhibited increased lipid uptake and deposition as well as increased infiltration into the lesion site, which resulted in an enlarged necrotic core compared to control *Apoe^−/−^* mice (114). USP9X is a factor that suppresses lipid uptake in macrophages by targeting SR-A1 for degradation upon ox-LDL contact, thereby reducing SR-A1 internalization and foam cell formation. Conversely, USP9X genetic ablation or pharmacological inhibition promotes SR-A1 internalization and foam cell development (114).

As expected, cholesterol transporters, ABCA1 and ABCG1, are also important targets through which foamy macrophages influence atherosclerosis progression (115). A recent study has shown that YXTMD, a traditional Chinese decoction, effectively increases cholesterol efflux by activating PPARγ-LXR-ABCA1/ABCG1 pathway in foamy macrophages and attenuates atherosclerosis in *Apoe^−/−^* mice (116). Similarly, disruption of LXR signaling in Mφ can intensify both lipid accumulation and inflammatory activation, dramatically increasing atherosclerosis and plaque inflammation (117).

### 5.3 Foamy macrophages in human plaques

In humans, plaque macrophages, are not a homogenous cell type but rather comprise multiple subsets with diverse functional roles ranging from inflammatory mediators to plaque-stabilizing cells. To this extent, immunohistochemical analyses of human carotid atherosclerotic plaques (*n* = 27) also confirmed the presence of both M1-like and M2-like macrophages (118). Thus, carotid artery plaques from symptomatic coronary artery disease (CAD) patients were enriched with pro-inflammatory M1-like macrophages, whereas plaques from asymptomatic patients contained more anti-inflammatory M2-like macrophages (119). In a 2023 study, Patterson et al. analyzed symptomatic and asymptomatic carotid endarterectomy samples (70), identifying 19 distinct myeloid cell populations. Monocyte and macrophage clustering revealed four subsets expressing the myeloid markers CD14 and PTPRC, each with unique gene signatures ranging from inflammatory genes (IL-1β, NLRP3) to lipid-processing genes (FABP5, LGALS3). TREM2 expression was exclusive to foamy macrophages in human plaques (35), whereas foamy macrophage–associated genes (CD9, LPL, FABP4) were predominantly observed in asymptomatic plaques, suggesting that these foam cells may play a stabilizing role (7).

## 6 Inflammatory phenotype of foamy macrophages and possible therapeutic potential

The inflammatory profile of foamy macrophages remains under active investigation, and different receptors may determine this process. Recent studies have presented contrasting findings on whether foamy macrophages predominantly exhibit pro- or anti-inflammatory characteristics. For example, myeloid-specific deletion of TREM2 markedly attenuates plaque progression (35). The same authors have, however, also demonstrated that using a TREM2-specific agonist (AL002a) increases lipid uptake and cholesterol efflux, driving an anti-inflammatory phenotype that leads to plaque stability (120). This finding is in agreement with another recent manuscript, which suggests that TREM2 activation decreased the necrotic core and improved macrophage efferocytosis in late atherosclerosis (36). Transcriptomic analysis by Kim et al. showed that, in murine atherosclerotic models, non-foamy macrophages are more pro-inflammatory than foamy macrophages and substantially contribute to disease progression (7). Moreover, non-foamy macrophages not only have higher expression of genes involved in pro-inflammatory responses such as *IL-1β*, *Tnf*, *Ccl2*, *Cx3cr1*, *Nlrp3*, and *TREM1* (7, 64) but also atherosclerosis-associated genes such as *Egr1*, *Nlrp3*, *Cebpb* (56, 121, 122). In contrast, foamy macrophages upregulate genes involved in lipid metabolism rather than genes involved in inflammation (103, 123). In addition to differences in the inflammatory signature of foamy and non-foamy macrophages, they are also distributed differently in the atherosclerotic plaques. Non-foamy macrophages expressing inflammatory phenotype are predominantly present in aortic adventitia, while foamy macrophages are homed in aortic intima in atherosclerotic plaques and mainly express lipid metabolism gene signature (7, 124). On the other hand, another study has demonstrated upregulation of TREM2 expression in foamy macrophages in atherosclerotic plaques in *Apoe^−/−^* mice fed on high-fat diet (110). Using a *TREM2/Apoe* double-KO model, others demonstrated that atherosclerotic lesions were significantly smaller, with fewer foamy cells and lower lipid load compared to *Apoe^−/−^* mice alone (110). A better characterization of foamy macrophage function may help define their role in atherosclerosis progression and identify effective therapeutic targets. Interestingly, Dib et al. identified a transition in lipid-associated macrophages from a TREM2-expressing, more reparative phenotype to a TREM1-expressing, pro-inflammatory state in human atherosclerotic plaques. While TREM2 promotes lipid handling and limits inflammation, TREM1 amplifies inflammatory signaling, driving plaque progression and instability (125).

To this extent, our group and others also recently described another receptor that regulates macrophage function and atherosclerosis progression: Olfactory Receptor 2 (Olfr2) (96). Our group has previously demonstrated that macrophages can express Olfr2 and its activation through octanal, a known ligand of Olfr2, induces cyclic adenosine monophosphate (cAMP), Calcium flux, ROS production, and Nlpr3 inflammasome activation to produce IL-1β in BMDMs. Thus, Olfr2 depletion has been shown to reduce atherosclerosis in mice (126). However, the actual physiological mechanism of Olfr2 *in vivo* has not yet been comprehensively described and it is an active area of investigation. Recently by using mass cytometry, we identified that Olfr2-expressing macrophages account for at least 30% of aortic macrophages and are characterized by the high expression of CD64, CCR2, and CD11c (127). Furthermore, Gene Set Enrichment Analysis (GSEA) using defined gene signatures revealed that these Olfr2 + macrophages were enriched with MDMs, aortic resident macrophages (Mac^AIR^), and a TREM2 + subsets (127). Approximately half of the Olfr2 + macrophages are foamy, as identified by the fluorescent lipid probe BODIPY, and produce elevated levels of IL-6 and TNF compared to BODIPY + foamy macrophages lacking Olfr2. These findings suggest that Olfr2 + macrophages can be both foamy and pro-inflammatory, offering a potential means to distinguish different foamy macrophage phenotypes in atherosclerosis (127).

Targeting these pathways with inhibitors of Olfr2 or agonists such as AL002a for TREM2 could be valuable in modulating foam macrophage phenotypes, promoting plaque stability, ultimately improving patient outcomes, and reducing complications. However, further understanding of the physiological mechanisms controlling these pathways, and possibly others, is essential to achieve possible clinical applications. Moreover, to determine the translational impact of the TREM2/TREM1, and Olfr2 therapies, the development of induced pluripotent stem cells derived *in-vitro* vascular organoids may be beneficial to screen drugs that are more effective in lowering atherosclerosis disease burden in humans.

## 7 Conclusions and outlook

In conclusion, foamy macrophages emerge as key players in atherosclerosis, exhibiting both protective and pathological roles according to their origin, stage of disease progression, and receptor expression profiles. While these foam cells can initially help clear modified lipids and maintain arterial homeostasis, their prolonged accumulation and phenotypic transitions driven by differential regulation of receptors such as TREM2, TREM1, and possibly Olfr2, together with compromised cholesterol uptake and export pathways (e.g., LXR, ABCA1, ABCG1), ultimately fuel chronic inflammation and promote plaque instability. Recent single-cell analyses highlight the functional heterogeneity of foamy macrophages, revealing subsets that either mitigate or exacerbate disease. Targeting specific pathways that govern modified-LDL uptake, cholesterol efflux, and pro-inflammatory signaling, potentially through modulation of TREM2, TREM1 and possibly others, may offer a promising avenue to rebalance macrophage function and improve plaque stability. Future work to pinpoint these regulatory mechanisms and to harness them for safe, effective interventions could advance the therapeutic landscape for atherosclerotic cardiovascular disease prevention and cure.
